# Splenic abscess in cancer chemotherapy

**DOI:** 10.1186/s13104-015-1655-1

**Published:** 2015-11-11

**Authors:** Essadi Ismail, Rachid El Barni, Mohamed Lahkim, Redouane Rokhsi, Elmehdi Atmane, Abdelghani El Fikri, Rachid Bouchama, Abdessamad Achour, Mohamed Zyani

**Affiliations:** Medical Oncology Unit, IBN SINA Military Hospital, Marrakesh, Morocco; General Surgery Unit, IBN SINA Military Hospital, Marrakesh, Morocco; Internal Medicine Unit, IBN SINA Military Hospital, Marrakesh, Morocco; Radiology Unit, IBN SINA Military Hospital, Marrakesh, Morocco

**Keywords:** Splenic abcess, Cancer Treatment, Complication

## Abstract

**Background:**

Splenic abcess is an uncommon complication for cancer treatment. It occurs more frequently in immunocompromised patients. They are characterized by high mortality. The classic triad (fever, pain of the left hypochondrium, and sensitive mass left) is only present in one-third of cases the clinical spectrum ranging from no symptoms to events such as fever, nausea, vomiting, weight loss, abdominal pain left, splenomegaly. Treatment options are limited, but must be discussed and adapted to the patient profile.

**Case presentation:**

We report the case of a 62-year-old Arabic male, diagnosed with metastatic lung adenocarcinoma, who, after several cycles of chemotherapy, presented symptoms and signs of splenic abcess.

**Conclusion:**

Splenic abcess is rare situation, which must be actively researched, to have access to an optimal therapeutic approach.

## Background

The splenic abscess is a rare condition affecting immunocompromised patients [1]. Without the contribution of imaging, it is difficult to diagnose and often fatal if left untreated. It is associated with high mortality (47–100 %) [1, 2]. Recent years, there has been more frequent diagnoses, partly because of the increased incidence of immunocompromised patients, and on the other hand to the availability of more efficient complementary tests such as computed tomography (CT) [3]. The use of cancer chemotherapy is often a situation that promotes immunodepression [3, 4]. In this context, this article reports a case of splenic abscess diagnosed in a cancer patient treated with antimitotic chemotherapy. The goal is to attract the attention of practitioners on this relatively rare complication, which is often under-diagnosed, and propose a therapeutic approach through a review of the literature.

## A case presentation

**Fig. 1:**
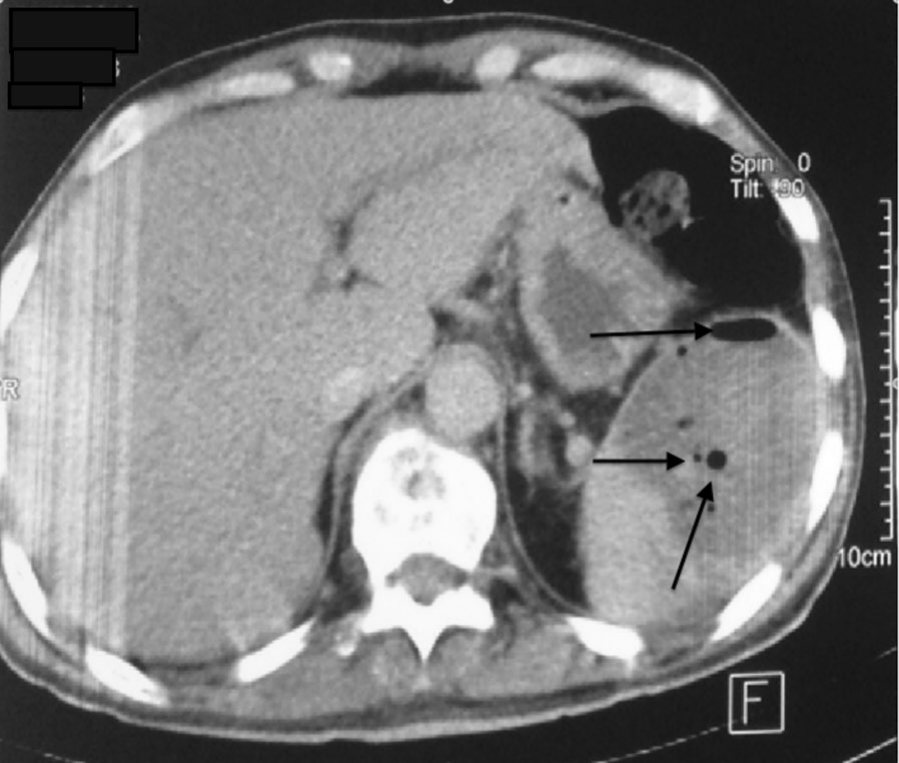
Splenic collection with air bubbles

**Fig. 2:**
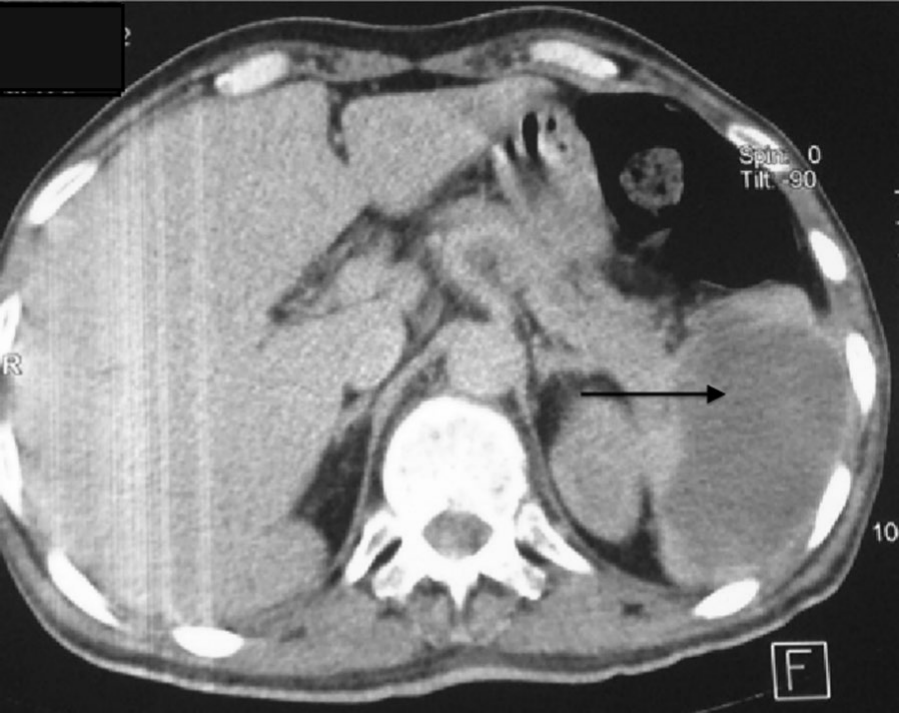
Splenic collection 9 cm big axis

**Fig. 3:**
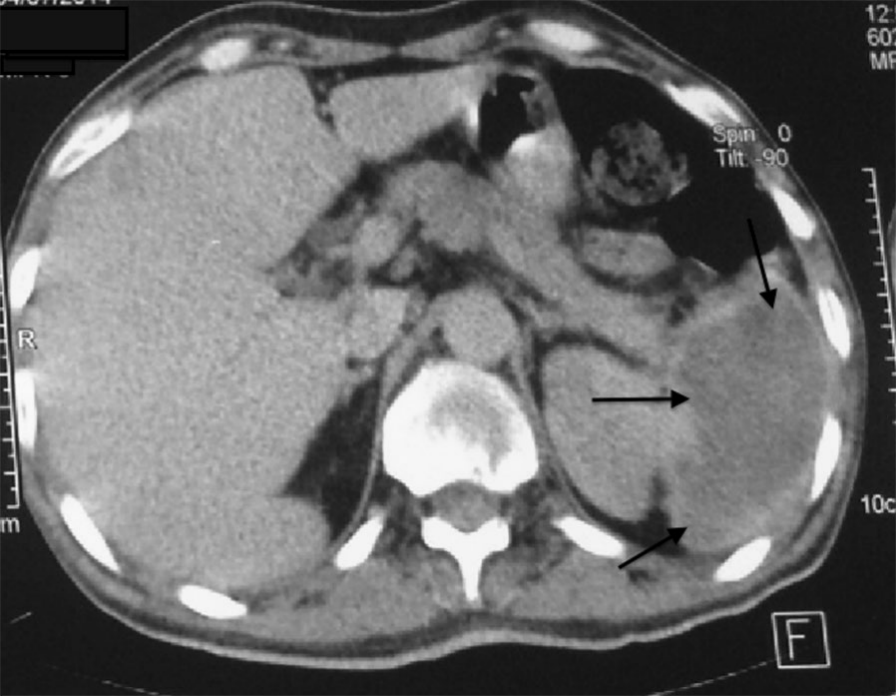
Collection of the lower pole of the spleen

A 62-year-old Arabic male admitted to our medical oncology department for management of lung adenocarcinoma. The initial workup included a CT scan of the body and a bone scan, which revealed lung, axillary and supraclavicular lymph nodes metastases, chemotherapy with an Bevacizumab–Paclitaxel and Carboplatin regimen was initiated: Paclitaxel 220 mg/m^2^–Carboplatin area under the curve (AUC) 6 and Bevacizumab 15 mg/kg. His radiological and clinical evaluation after two courses of treatment showed a stable disease, with significant improvement in quality of life. After his fourth cycle of chemotherapy, he presented to our emergency department in the context of febrile neutropenia with neutrophil 600/mm^3^ and a fever of 40 °C. A broad spectrum antibiotic therapy was introduced, combining amoxicillin–clavulanic acid and ciprofloxacin after various bacteriological samples. After 48 h of antibiotic therapy, neutrophils are recovered to 1500/mm^3^, but the fever did not improve. C-reactive protein remained high at 342 mg/l. A thoracoabdominal CT has been done looking for a deep collection. It showed the presence of a splenic abscess, with a major axis of 9 cm, and the presence of air bubbles certifying an anaerobic infection (Figs. 1, 2, 3). The patient underwent a emergency splenectomy, with bacteriological analysis of the pus that was chocolate colored. The offending organism was a Pyocianique, sensitive to ceftriaxone and gentamicin. Antibiotics adapted to the antibiogram was introduced, with a marked improvement in the general condition of the patient, who was discharged after 10 days of surgery. Histopathological study was also performed. It has objectified a nonspecific inflammatory infiltrate without malignant cells. Vaccination against *Pneumococcus*, *Haemophilusmeningitis* and seasonal influenza was conducted 2 weeks after splenectomy.

## Discussion

Spleen abscesses occur more frequently in immunocompromised patients [1]. Infection with human immunodeficiency virus (HIV), the use of intravenous drugs, diabetes, immunosuppressive treatments, hepatic parenchymal disease (notably chemoembolization for hepatocellular carcinoma) or pancreatic neoplasia and alcoholism are more comorbidities reported [4, 5]. In our case, the patient was followed for metastatic lung cancer receiving chemotherapy, and admitted in an array of febrile neutropenia. The pathophysiology of splenic abscesses based on several theories: hematogenous theory where the spleen is infected during severe sepsis; the intrinsic theory where the infection occurs in an altered structure or function such as splenic infarction or splenic hematoma and extrinsic theory where the spleen is contaminated by an infection of the neighborhood [6, 7]. The first two theories can be accepted in our patient, especially since it was in anticancer chemotherapy. The bacteria most frequently involved are *Staphylococcus aureus*, *Streptococcus*, Enterobacteriaceae especially the salmonellosis and anaerobic [8]. In the case of our patient bacteriological study found a pyocianique certifying the nature of nosocomial infection probably spread through blood, when handling the implantable chamber. The symptoms of splenic abscess is polymorphic [9, 10]. Febrile painful splenomegaly is inconstant [7, 11]. Similarly, the peritonitis is a rare circumstance discovery. In some cases, symptoms boils down to infectious syndrome or persistent impairment of the general condition and the diagnosis is imaging [12]. In our patient, the infectious syndrome was at the forefront which delayed the diagnosis of 48 h. CT has the same reliability as ultrasound for the diagnosis of splenic abscess [10]. The treatment of splenic abscesses based on empiric antibiotic therapy secondarily adapted to bacteriological results more or less associated with ultrasound-guided puncture, Percutaneous drainage or splenectomy [13, 14]. Percutaneous drainage has the advantage of shortening the duration of hospitalization, preventing peritonitis due to rupture of the abscess and preserve the splenic parenchyma with cure rates ranging from 70 to 100 % [13, 14]. Conservative treatment seems to be more effective in the unique collections, with a thin wall, or in bad general condition of the patient [12–14]. Splenectomy remains a good indication if partitioned or multiple abscesses in case of failure of percutaneous treatment or in case of complicated abscesses [12]. Splenectomy was offered to our patient because of his land degradation. It is also clear that the total splenectomy puts them at increased risk of death from severe infections post-splenectomy with organ failure or overwhelming post splenectomy infection (OPSI) [15, 16]. It is necessary to prevent these infections in splenectomized by vaccination against *Pneumococcus*, *Haemophilus* and meningococcal and pneumococcal a long-term antibiotic prophylaxis with penicillin V [16].

## Conclusion

Here we report the case of a patient presenting with splenic abcess, with a large collection having appeared under cancer chemotherapy. This infectious event is very uncommon, making it almost impossible to perform prospective clinical trials specifically designed to compare different treatment approaches. Thus, only a greater awareness of the problem along with a more accurate and timely diagnosis, will lead to choosing the best therapies suitable to the specific patient and, in turn, improve overall prognosis.

## Consent

Written informed consent was obtained from the patient for publication of this case report and any accompanying images.

## Abreviations

AUC: area under the curve
CT: computed tomography
HIV: human immunodeficiency virus
OPSI: overwhelming post splenectomy infection
